# Neonicotinoid Insecticide Residues in Surface Water and Soil Associated with Commercial Maize (Corn) Fields in Southwestern Ontario

**DOI:** 10.1371/journal.pone.0118139

**Published:** 2015-02-24

**Authors:** Arthur Schaafsma, Victor Limay-Rios, Tracey Baute, Jocelyn Smith, Yingen Xue

**Affiliations:** 1 Department of Plant Agriculture, University of Guelph, Ridgetown Campus, Ridgetown, ON, Canada; 2 Ontario Ministry of Agriculture Food and Rural Affairs, Ridgetown, ON, Canada; Ghent University, BELGIUM

## Abstract

Neonicotinoid insecticides have come under scrutiny for their potential unintended effects on non-target organisms, particularly pollinators in agro-ecosystems. As part of a larger study of neonicotinoid residues associated with maize (corn) production, 76 water samples within or around the perimeter of 18 commercial maize fields and neighbouring apiaries were collected in 5 maize-producing counties of southwestern Ontario. Residues of clothianidin (mean = 2.28, max. = 43.60 ng/mL) and thiamethoxam (mean = 1.12, max. = 16.50 ng/mL) were detected in 100 and 98.7% of the water samples tested, respectively. The concentration of total neonicotinoid residues in water within maize fields increased six-fold during the first five weeks after planting, and returned to pre-plant levels seven weeks after planting. However, concentrations in water sampled from outside the fields were similar throughout the sampling period. Soil samples from the top 5 cm of the soil profile were also collected in these fields before and immediately following planting. The mean total neonicotinoid residue was 4.02 (range 0.07 to 20.30) ng/g, for samples taken before planting, and 9.94 (range 0.53 to 38.98) ng/g, for those taken immediately after planting. Two soil samples collected from within an conservation area contained detectable (0.03 and 0.11 ng/g) concentrations of clothianidin. Of three drifted snow samples taken, the drift stratum containing the most wind-scoured soil had 0.16 and 0.20 ng/mL mainly clothianidin in the melted snow. The concentration was at the limit of detection (0.02 ng/mL) taken across the entire vertical profile. With the exception of one sample, water samples tested had concentrations below those reported to have acute, chronic or sublethal effects to honey bees. Our results suggest that neonicotinoids may move off-target by wind erosion of contaminated soil. These results are informative to risk assessment models for other non-target species in maize agro-ecosytems.

## Introduction

Neonicotinoids are a class of systemic insecticides, which are absorbed and translocated throughout the plant and provide in-plant protection from pests for a period during plant establishment [1]. The neonicotinoid class of insecticides were adopted to replace older carbamate and organophosphate chemistries considered to be more hazardous to applicators and other non-target organisms particularly mammals [2]. Neonicotinoids may be applied as seed treatments, foliar sprays, soil drenches, granules, injection or through—irrigation systems [2]. The variety of methods for their application, along with their systemic properties and low toxicity to mammalian vertebrates has resulted in wide-spread adoption for crop protection [2,3]. In particular, their convenience as seed treatments has resulted in a shift from their prescriptive use in response to pest scouting, to prophylactic use as insurance against pest damage [3]. Currently-seven neonicotinoids, clothianidin, imidacloprid, thiamethoxam, dinotefuran, acetamiprid, thiacloprid and nitenpyram, are used in agricultural production [4]. Imidacloprid was the first neonicotinoid registered on field crops in Canada in 2001 [5]. Presently, three of these, clothianidin, thiamethoxam and imidacloprid are registered for use on almost all major field crops in Canada. Clothianidin and thiamethoxam are the neonicotinoids most commonly used in southwestern Ontario. This group of insecticides dominates the seed treatment market in Ontario—with over 99% of maize (corn), 60–80% of soybean, 95% of dry bean, 25% of winter wheat and 100% of canola crop areas planted with a neonicotinoid seed treatment in 2013 [5].

Pollinator Colony Collapse Disorder (CCD) is a major issue facing apiculture globally [6–13]. Many interacting and confounding factors are debated to cause honey bee (*Apis mellifera* L.) decline, including parasites, disease, pesticide exposure, habitat loss, and climate change, with no single factor standing out as the primary cause [8,14–24]. Historically most acute bee poisonings arising from pesticide use have occurred from exposure to broadcast applications of insecticides on plants on which bees are foraging [25]. Recently, coincident with the widespread adoption of neonicotinoid insecticides as seed dressings for field crops and the introduction of pneumatic maize planters, acute bee mortality near planting time has been observed in Europe [26–31], the United States [32] and in Canada [33,34]. This has been attributed to exposure to neonicotinoid insecticide residues escaping from the planter’s vacuum exhaust [3,33–38]. Besides direct exposure to flying bees, these residues have the potential to contaminate flowers, pollen or other resources used by bees [32]. Neonicotinoid residues have been found in fresh pollen of plants [32,39–41], bee-collected pollen [–29,32,33,42], nectar [41], plant guttation fluid [43,44], and planter exhaust dust [26–32]. Neonicotinoid insecticides are water-soluble, making them candidates for surface water contamination [45–48] and have been shown to be persistent in water and soil under some environmental conditions [49].

Honey bees collect water for several purposes: to thermo-regulate their nest by evaporative cooling [–50], to dilute stored honey and glandular secretions to feed brood, and to maintain humidity in their nests for larval development [51]. Water is collected by individual foragers with some specialized to this task for long periods [52]. Water foraging bees can collect 44 μL of water/bee for each water-collecting flight [53]. Each bee in a group of 200 consumes about 11 μL of water daily at 35°C [54]. Neonicotinoid residues in surface water may also affect aquatic invertebrates and ecosystem health [45–47,55]. Currently there are only limited data available on neonicotinoid residues in water sources related to commercial maize production that may be used by honey bees, except a recent report of neonicotinoids detected in streams of an area of intense corn and soybean production of Midwestern United States [56]. We hypothesized that neonicotinoid residues occurring in water sources within and in close proximity to commercial maize fields are a source of exposure to honey bees. Our objective was to investigate the quantity, distribution and temporal dynamics of clothianidin and thiamethoxam in surface water related to maize production, as one consideration to inform the risk assessment of acute and chronic exposure of honey bees to neonicotinoid insecticide residues. Melted snow in the spring in Canada is also an important water source around agricultural fields. The average annual precipitation from 1981 to 2010 for the five counties where our sites are located (Essex, Chatham- Kent, Lambton, Middlesex and Elgin) ranged from 878 to 1012 mm. Average annual snowfall ranged from 79 to 166 mm as rainfall equivalent, which accounted for 9–16% of the average annual precipitation, respectively [57]. We have observed that drifted snow often contains soil scoured from fields during high winds and we hypothesized these soil residues could be contaminated by neonicotinoid insecticides possibly contributing to detectable residues in standing water around fields. For this study a fully validated liquid chromatography/tandem mass spectrometry (LCMS/MS) method is described for the simultaneous analysis of clothianidin and thiamethoxam in soil and water.

## Materials and Methods

### Ethics statement

These field studies did not involve endangered or protected species. No specific permits were required to access the field study sights and all were privately owned by cooperators for agricultural crop production, and visited by permission. Permission was obtained by Tracey Baute, Provincial Entomologist, Ontario Ministry of Agriculture Food and Rural Affairs. Under privacy law we are unable to publicly disclose the names or contact information of the cooperators without their permission. We have all this on record and access can be requested for verification or additional work in future by contacting Tracey by email (tracey.baute@ontario.ca).

### Chemicals and reagents

Certified clothianidin and thiamethoxam standards and their respective deuterium-labeled internal standard (clothianidin-d3 and thiamethoxam-d3) were obtained from Sigma-Aldrich (St. Louis, MO, USA; Pestanal class, purity ≥99.5%). LCMS grade methanol was obtained from JT Baker (Phillipsburg, NJ, USA). LCMS grade water and acetonitrile were obtained from OmniSolv (Billerica, MA, USA). Anhydrous magnesium sulfate and sodium chloride, sodium citrate tribasic dehydrate and sodium hydrogencitrate sesquihydrate were obtained from Sigma-Aldrich. N-hexane and formic acid were purchased from Acros Organics (Geel, Belgium, part of Thermo Fisher Scientific Inc.). Stock solutions were prepared by dissolving clothianidin and thiamethoxam standards to 1mg/mL and their respective deuterated internal standard to 100 ug/ml in acetonitrile in amber glass flasks. Mixed working standard solutions were prepared for these two neonicotinoids at 10 ng/ml and 100 ng/ml and their respective internal standard at 100 ng/ml in 1:1 methanol/water with 5mM formic acid. All solutions were stored in darkness at 2°C.

### Experimental fields

**Fig 1:**
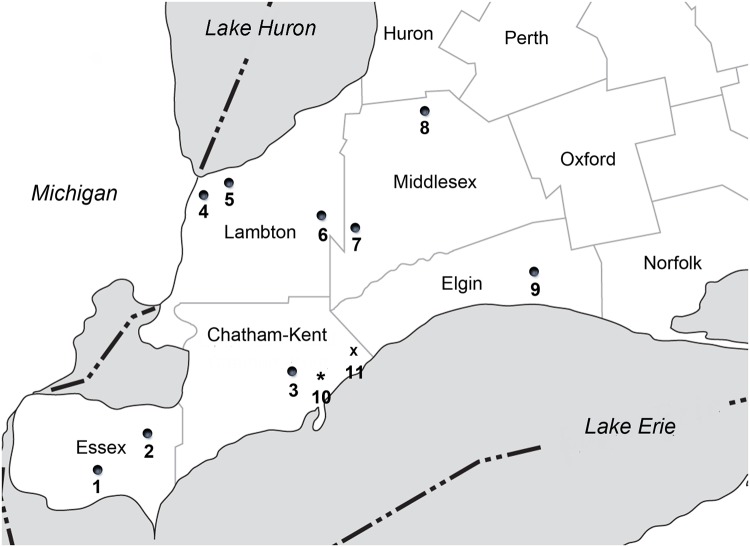
Map of subject field locations in 2013 (modified from the original by Cartographic Office, Department of Geography, University of Guelph). 1–9: maize fields and bee yards; 10: fields for snow drift samples; 11: blank soil taken.

As part of a larger study on honey bee exposure to neonicotinoid residues in maize fields, we enlisted nine farm cooperators, each contributing 2 commercial maize fields (minimum 20 ha in size, most 40 ha or larger), with both fields paired with one apiary within a 3-km radius of both fields. Each cooperator was located in one of 5 counties (Essex, Chatham- Kent, Lambton, Middlesex and Elgin) in southwestern Ontario (Fig. 1). Field characteristics are given in Table 1 and their GPS locations can be found in the S1 Table).

**Table 1:** Soil texture class and crop rotation and tillage history of subject maize (corn) fields.

Field	Soil texture class	Crop/Tillage 2010	Crop/Tillage 2011	Crop/Tillage 2012	Crop/Tillage 2013
1A	Clay loam	Soybeans/no till	Winter wheat/no till	Soybeans/no till	Maize/no till
1B	Clay loam	Soybeans/no till	Wheat/no till	Soybeans/no till	Maize/no till
2A	Silty clay loam	Maize/minimum till	Soybeans/no till	Soybeans/no till	Maize/minimum till
2B	Clay loam	Maize/minimum till	Soybeans/no till	Winter wheat/no till	Maize/minimum till
3A	Loam	Maize/conventional	Maize/conventional	Maize/conventional	Maize/conventional
3B	Sandy loam	Maize/conventional	Maize/conventional	Maize/conventional	Maize/conventional
4A	Silt loam	Soybeans/no till	Winter wheat/no till	Sugar beets/conventional	Maize/conventional
4B	Silt loam	Soybeans/no till	Sugar beets/conventional	Winter wheat/no till	Maize/conventional
5A	Loam	Maize/conventional	Soybeans/no till	Sugar beets/conventional	Maize/conventional
5B	Loam	Soybeans/no till	Winter wheat/no till	Sugar beets/conventional	Maize/conventional
6A	Silt loam	Soybeans/no till	Maize/conventional	Soybeans/no till	Maize/conventional
6B	Silt loam	Soybeans/no till	Maize/conventional	Soybeans/no till	Maize/conventional
7A	Silt loam	Maize/minimum till	Soybeans/no till	Soybeans/no till	Maize/conventional
7B	Silt loam	Maize/minimum till	Soybeans/no till	Soybeans/no till	Maize/conventional
8A	Silt	Maize/conventional	Soybeans/conventional	Winter wheat/no till	Maize/conventional
8B	Silt	Winter wheat/conventional	White pea Beans/conventional	Winter wheat/no till	Maize/conventional
9A	Silt loam	Maize/conventional	Maize/conventional	Maize/conventional	Maize/conventional
9B	Loam	Maize/conventional	Green beans/conventional	Seed wheat/conventional	Maize/conventional

### Sample collection

Sample fields and apiaries were surveyed weekly from 29 April to 28 June 2013 for water sources that bees could potentially visit. Field samples were categorized as being from “within” or “outside” of the subject fields where “within” field samples were collected from puddles of standing water within the perimeter of the field and “outside” samples were collected from puddles, ditches or field drainage outlets from within 0 to 100 m of the field perimeter. At apiaries numbered 1, 4, 5, and 7 puddles were sampled within 100 m from the beehives and water collected at apiary 9 was from a pond (10 m in diameter) within 10 m of the beehives. No water samples were found for collection at apiaries numbered 2, 3, 6, or 8. If multiple water samples of the same type (e.g. puddles within field) were collected from a field within the same sampling period these were considered repeated measures in space. However, it should be noted that the subject fields were large and that when multiple samples were taken from the same field, they were from different parts of these fields and most likely had different characteristics from one another. Approximately 100 ml of water was collected for each sample in a new 100 ml amber HDPE bottle (Fisher Scientific, Ottawa, ON) using a new 50 ml disposable syringe (Fisher Scientific, Ottawa, ON), placed in a cooler with freezer packs in until returned to the laboratory by the end of each sampling day to be stored at -20°C in complete darkness until analyzed using LC-MS/MS.

On the day of planting, soil grab samples were taken from the top 5 cm of the surface (representing the depth of seed placement zone) immediately before and immediately after planting. Ten subsamples were taken at random within each field, while walking in an M pattern across the field; sub-samples taken after planting were collected from the mid-point between two newly planted rows to avoid the width of the placement zone of treated seeds. Subsamples were obtained from conventionally tilled fields by scooping the appropriate amount by a hand that was protected by a virgin disposable nitrile glove (Fisher Scientific, Ottawa, ON) or from no-till fields using a disposable 100 mm × 15 mm Petri dish (Fisher Scientific, Ottawa, ON) where the surface soil was previously undisturbed. Each subsample was approximately 100 g; subsamples were pooled directly into a new plastic bag that was then placed into another plastic bag, and mixed thoroughly in the field. The sample was placed immediately into a dark picnic cooler containing freezer packs for transport back to the laboratory, followed by immediate dark and frozen storage (-20°C) until analysis.

Two samples of top soil (0–10 cm deep, 2 kg each) were collected from an old growth Carolinian forest located at Clear Creek Forest near Clearville, ON, approximately 0.5 km from the nearest surrounding agricultural land, to provide soil of the region for use as a blank sample for analytical method validation.

As an unplanned and preliminary study to determine if drifting snow is a potential contributor to off-site neonicotinoid residues, we took the opportunity to sample two snow drifts each located on the leeward side of two adjacent 40-ha fields near Ridgetown, ON. These two fields are typical in soil type and cropping practice of those found in the region, and occur somewhat central to region where the water samples were taken. A series of small to moderate snow events, (3–10 cm) each accompanied by high winds, occurring on frozen bare soil, resulted in large snow drifts on the edge of these two fields in January. Conveniently, these fields were similar in size, soil type, topography, cropping history, and history of neonicotinoid use. The leeward field had been tilled in the autumn and left bare, while the windward field had been planted with winter wheat without tillage. There were obvious alternating strata of clean and soiled snow in the drift profile.

The first sample was collected on 27 January in the windward field from a snow drift approximately 2 m in height and 3 m wide at the base located along the center of the eastern edge. This first sample was a composite of numerous small sub-samples, taken randomly through the vertical profile of the drift on 27 January to see if neonicotinoid residues could be detected. This field had a crop history of wheat in 2011 followed by maize in 2012 and soybeans in 2013, and had a variable soil type and topography. Winter wheat was planted again in the fall of 2013 with no tillage. All of the seed for these crops was treated with a neonicotinoid insecticide at the lowest recommended rate for each crop. All samples were taken with a snow shovel that had been cleaned by thoroughly scrubbing with the visibly cleanest snow from the drift the sample was to be taken from. After neonic residues were confirmed in this first sample, the second two samples were taken on 30 January, each consisting of a 0.027-m3 snow cube one from the same drift as for 27 January and the second taken from a similar drift with similar dimensions on the leeward side of the neighbouring field across the road to the east. For each drift a vertical face was dug out to the ground level to expose the layers of drifted snow. A single sample was taken from the thickest layer of soiled snow in the drift from each field. We selected the same layer from each drift assuming they were deposited during the same drifting event. These samples were collected inside a new black plastic bag fit into a bucket, taken immediately to the laboratory and allowed to melt in darkness at room temperature for 48 h before extraction. There was no replication.

### Neonicotinoid extraction

Clothianidin and thiamethoxam were extracted using the QuEChERS (quick, easy, cheap, rugged and safe) procedure [58] without a solid phase extraction [59] by placing 10 ml of water or melted snow or 10 g of soil into a 50 mL polypropylene disposable centrifuge tube, adding 20 mL acetonitrile, vortexing for 1 min followed by addition of 4 g magnesium sulphate, 1 g sodium chloride, 1 g sodium citrate tribasic (SCT) and 0.5 g sodium hydrogencitrate sesquihydrate (SHS) and shaking thoroughly for 1min. Each sample was then centrifuged for 5 min at 3000 rpm in a table-top Thermo IEC Central CL2 centrifuge (Milford, MA, USA). A 4-mL aliquot of the resulting acetonitrile supernatant was transferred to a 5 mL glass test tube containing 20 μL of 100 ng/ml mix internal standard solution followed by evaporation to dryness using a Pierce Reacti-Therm III heating module (Rockford, IL, USA) at 40°C under gentle filtered air stream in darkness. The final residue was reconstituted in 1 mL of 1:1 methanol/water (v/v) containing 5 mM formic acid (dilution solvent), vortexed for 2 min and transferred to a 2 mL amber glass auto-sampler vial for LC-MS/MS analysis. Moisture content was determined by oven drying soil samples until weight stabilized, and comparing the dry versus wet mass. Concentration in soil was corrected and expressed in terms of dry soil mass. Melted snow samples were poured into 10 L autoclavable polypropylene storage bottles and homogenized over 5 min using a magnetic stir bar. A total of 4 subsamples were collected into 250 mL new Nalgene bottles under continuous stirring. All snow samples were analysed within 48 hr of collection using a 10 mL aliquot of each subsample giving a total of four replicates per sample.

### LC-MS/MS detection

Clothianidin and thiamethoxam determinations were made by injecting a 50 μL aliquot of extract into a 150 mm Gemini C18 reverse phase column (Phenomenex, Torrance, CA) with an Agilent 1100 Series HPLC (Agilent Technologies, Santa Clara, CA, USA). The peak off the HPLC was introduced to an Ionics EP 10+ modified API 365 triple quadruple mass spectrometer (AB SCIEX, Concord, ON) system equipped with an electrospray ionization source (ESI-MS/MS). The chromatographic separation was performed using a gradient program (25 min) with a binary mobile phase consisting of A (methanol and 5 mM formic acid) and B (water and 5 mM formic acid) at a flow-rate of 1 mL/min [60]. The source gas temperature was set to 550°C, nitrogen curtain gas 80 psi, nebulizer gas 8, collision gas 2, and ionization voltage 5000 V. Nitrogen gas was produced by a dedicated Parker Balston Source LCMS-5000 Tri Gas Generator (purity > 99%) and was used as the curtain, drying and collision gases (Haverhill, MA, USA). The gradient began with 25% A and ramped linearly over 15 min to 95% A, these conditions were held for 4 min and finally for a 6 min equilibration for resetting to initial conditions of 25% A. All parameters used in the detection and quantitation were obtained via direct infusion of individual analytes (10 ng/μL solutions in 1:1 methanol/water with 5 mM formic acid) into the ESI-MS/MS at 10 μL/min using a Fusion 100 infusion pump (Chemyx Inc, Stafford, TX, USA) fitted with a 500 μL Gastight 1750 syringe (Hamilton, Reno, NV, USA). Each compound was analysed in positive ion polarity mode using a multiple reaction monitoring (MRM) procedure with one precursor ion (Q1) and two product ions (Q3). The most intense peak in Q3 was used for quantification (quantifier ion) and the second peak for confirmation (qualifier ion). MRM parameters were optimized by selecting collision energy and de-clustering, focusing and cell exit potentials producing the most intense response of the precursor and product ions for each corresponding analyte (Table 2). MRM transitions obtained for clothianidin, thiamethoxam and clothianidin-d3 were in agreement with values reported by Tanner and Czerwenka [60], while transitions found for thiamethoxam-d3 were 295.1 m/z for Q1, and 184.1 and 214.1 m/z for Q3 (Table 2).

**Table 2:** Optimized multiple-reaction monitoring (MRM) conditions used for the LC-ESI-MS/MS analysis of clothianidin, thiamethoxam and their respective deuterium-labeled internal standards (IS).

Analyte	R_t_ ^A^	Precusor ion	DP^B^	FP^C^	Productions^D^	CE^E^	CXP^F^
	(min)	(*m/z*)	(V)	(V)	(*m/z*)	(V)	(V)
Clothianidin	9.8	250.1 [M+H]^+^	81	252	131.8/**168.9**	20/17	19/20
Clothianidin-d3 (IS)	9.8	253.1 [M+H]^+^	81	252	132.0/**172.0**	20/17	19/20
Thiamethoxam	8.0	292.1 [M+H]^+^	100	306	181.1/**211.1**	29/17	21/25
Thiamethoxam-d3 (IS)	8.0	295.1 [M+H]^+^	100	306	184.1/**214.1**	29/17	21/25

^A^Retention time,

^B^declustering potential,

^C^focussing potential,

^D^quantifier ion/**qualifier ion**,

^E^collision energy,

^F^cell exit potential

**Fig 2:**
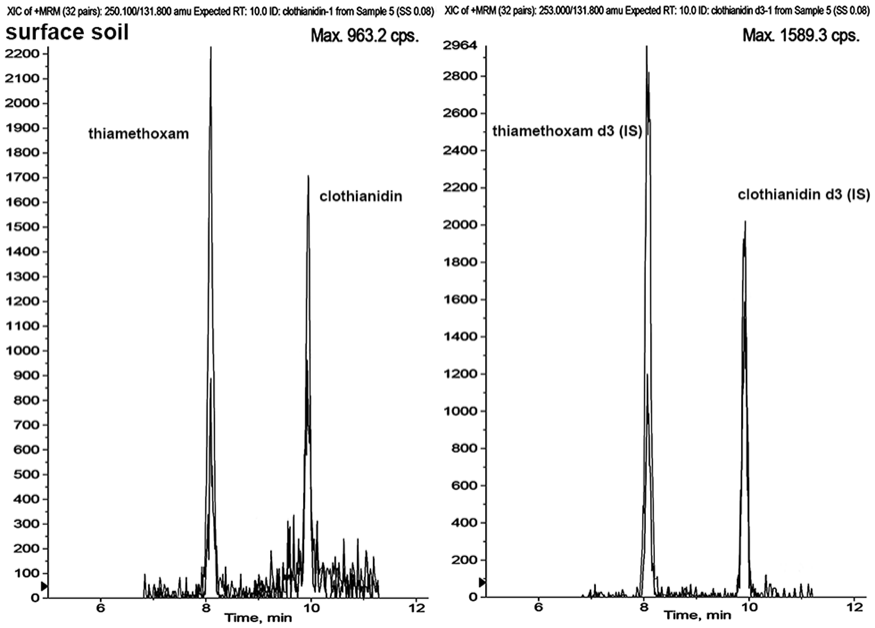
LC-ESI (+)-MS/MS MRM chromatograms of a soil sample spiked with 0.04 ng/g of clothianidin and thiamethoxam and 1 ng/g clothianidin-d3 and thiamethoxam-d3 internal standards.

For soil analyses, blank samples used in the recovery and calibration experiments were obtained as described previously. Each sample was divided into 3 subsamples and analysed separately for the presence of the target compounds. The subsample with the lowest concentration of clothianidin was used as the blank matrix. For analysis of water and melted snow, filtered municipal well water was used for blank samples. Recovery tests were performed in triplicate by spiking homogenized 5 g soil or 5 ml water blank samples with the appropriate volume of analytical standards at three concentrations; 0.10, 1.00 and 10.00 ng/g for soil; and 0.05, 0.50 and 5.00 ng/ml for water. Spiked samples were left to equilibrate for 3 d at 40°C in darkness followed by a standard extraction procedure and analysis. In this study, to compensate for variation of signal intensities due to matrix effect [61] commercially available deuterium analogs (clothianidin-d3 and thiamethoxam-d3) were used as stable isotopically labeled internal standards (IS, Fig. 2). Clothianidin and thiamethoxam concentrations were determined using matrix-matched calibration curves at 9 concentrations from 0.06 to 4.00 ng/mL in addition to a double blank (matrix extract) and a blank (matrix extract with their corresponding IS) sample.

LC-ESI-MS/MS system control, data acquisition, processing, peak-area integration, linear regression and final quantification were performed using Analyst 1.4.1 software (AB Sciex, Concord, ON). The linearity of the calibration curve was determined from the linear regression analysis of the peak area (height) ratios of analyte/IS versus analyte concentrations. Detection and quantification limits (LOD and LOQ) were based on estimating the contribution of the background noise signal found in the blank matrix extract. LOD and LOQ were calculated as the mean peak height (n = 4) that can be detected with reasonable certainty using the mean height of the noise signal plus 3 and 10 times standard deviation, respectively [62].

Soil samples collected from Clear Creek Forest and snow drift samples were analysed further for an additional 5 neonicotinoids (imidacloprid, thiacloprid, acetamprid, dinotefuran and nitempyram) and 3 herbicides (metolachlor, atrazine and imazethapyr) using the method described above. LOD, LOQ and average percentage of recovery of 3 spiked samples prepared in triplicate (0.10, 1.00 and 10.00 ng/g) are shown in Tables 3 and 4 for soil dry weight and melted snow, respectively.

**Table 3:** Determination of 7 neonicotinoids and 3 herbicide residues (ng/g; dry weight) using QuEChERS sample preparation and LC-ESI(+)-MS/MS analysis of two soil samples collected from a conservation forest of old growth Carolinian species.

Sample	clothianidin	thiamethoxam	imidacloprid	thiacloprid	acetamprid	dinotefuran	nitempyram	metolachlor	atrazine	imazethapyr
Sample 1	0.03	ND	ND	ND	ND	0.01	0.01	ND	0.04	ND
Sample 2	0.11	ND	0.01	ND	ND	ND	0.01	ND	0.05	ND
LOD	0.02	0.02	0.01	<0.01	0.01	0.02	0.09	<0.01	<0.01	<0.01
LOQ	0.06	0.04	0.05	0.01	0.04	0.05	0.21	0.01	0.01	0.01
% Recovery*	112.9	90.8	78.8	51.9	87.7	83.8	48.0	80.9	119.1	127.8

Clear Creek Conservation Area, Chatham-Kent, ON 2013.

* mean recovery of 3 spiked levels prepared in triplicate (0.10, 1.00 and 10.00 ng/g)

**Table 4:** Determination of 7 neonicotinoids and 3 herbicide residues (ng/ml of melted snow) using QuEChERS sample preparation and LC-ESI(+)-MS/MS analysis in three snow drift samples collected in January 2014 from the edge of two harvested maize fields near Ridgetown, ON (mean of four subsamples).

Sample	n	clothianidin	thiamethoxam	imidacloprid	thiacloprid	acetamprid	dinotefuran	nitempyram	metolachlor	atrazine	imazethapyr
Vertical drift composite in field 1	4	0.02	0.01	ND	0.01	0.01	0.02	ND	0.13	0.03	ND
Heavily soiled drift stratum in field 1	4	0.16	0.01	0.01	ND	0.01	0.02	ND	0.10	0.03	ND
Heavily soiled drift stratum in field 2	4	0.20	0.01	0.01	0.01	ND	0.02	ND	0.17	0.02	ND
LOD		0.02	0.01	0.01	<0.01	0.01	0.02	0.07	0.01	<0.01	<0.01
LOQ		0.05	0.02	0.03	0.01	0.02	0.05	0.16	0.02	0.01	0.01
*% Recovery		92.0	89.2	43.7	50.5	96.3	51.0	70.7	49.6	115.4	43.9

* Mean recovery of 3 spiked levels prepared in triplicate (0.05, 0.50 and 5.00 ng/ml);

n: number of subsamples analyzed

### Analytical performance

The validated LC-ESI-MS/MS method for simultaneous detection of both compounds showed good linearity for calibration curves (r >0.989) (Table 5) and repeatability expressed as relative standard deviations (<15%). LOD and LOQ were 0.017 and 0.037 ng/mL in water and 0.023 and 0.063 ng/g in soil, respectively, for clothianidin and 0.004 and 0.011 ng/mL in water and 0.017 and 0.045 ng/g in soil, respectively, for thiamethoxam. Mean recovery for all spiked samples was 92% and 112.9% for clothianidin and 89.2% and 90.8% for thiamethoxam in water and soil, respectively. Higher recovery rates for clothianidin were observed in the sample with the lowest spiked amount in soil due to a chromatographic peak from target compound found in the blank. All values were corrected for the recovery rate of each compound.

**Table 5:** Linearity of calibration curves, limits of detection and quantification, and recovery at 3 spiked levels of clothianidin and thiamethoxam in water (ng/ml) and soil (ng/g) samples.

	Linearity		LOD	LOQ	% Recovery		
	r	n			Spiking levels*		
Water (ng/ml)					0.05	0.50	5.00
Clothianidin	0.999	9	0.017 (8.5)	0.037 (6.7)	74.5 (14.2)	106.2 (8.2)	94.5 (2.3)
Thiamethoxam	0.998	9	0.004 (5.5)	0.011 (4.0)	103.0 (13.2)	81.5 (7.8)	83.2 (4.8)
Soil (ng/g)					0.10	1.00	10.00
Clothianidin	0.989	8	0.023 (4.3)	0.063 (0.2)	173.9 (14.8)	75.0 (10.8)	89.9 (9.5)
Thiamethoxam	0.999	7	0.017 (11.7)	0.045 (2.2)	94.2 (14.5)	88.1 (15.0)	90.1 (2.9)

Matrix-matched calibration curve was employed to determine limits of detection (LOD) and quantitation (LOQ).

*Analyte spiked at 3 levels and equilibrated for 3 days at 40°C before extraction. Number of replicates at each spiking level = 3. The values in brackets indicate the %RSD.

### Statistical analysis

Because clothianidin and thiamethoxam are the neonicotinoids used most commonly in southwestern Ontario, and clothianidin is a metabolite of thiamethoxam, we report the total quantity of clothianidin and thiamethoxam to represent the neonicotinoid residue, except when specifically noted. All statistical analyses were performed using SAS v. 9.4 (SAS Institute, Cary, NC). Water samples collected from “within”, “outside”, or from apiaries were grouped into four discrete sampling periods (Pre-plant (1–2 weeks before planting), 1–3, 4–5, and 6–7 weeks post-planting) to accommodate variations in sampling dates and differences in rainfall events at different locations and to allow comparisons of residues before and after fields were recharged with new neonicotinoid applications. The total neonicotinoid concentration in water data was subjected to log_10_ transformation to meet the assumptions of analysis of variance. Differences between the water sampling categories within each sampling period were analyzed using PROC MIXED with repeated measures where sample location (“inside”/”outside”/”apiary”) was the fixed effect, field was a random effect, and sample category was the repeated measure sampled from subject field. To ensure that assumptions of normally distributed residuals and homogeneous error variance were met, PROC UNIVARIATE was used to test residuals. The Shapiro-Wilk statistic was used to test residuals for normal distribution and studentized residuals were calculated to test for outliers. The α level for statistical significance was set at 0.05 for all analyses. For the analysis of neonicotinoid concentration in soil data were subjected to log_10_ transformation to meet the assumptions of normal distribution. Again PROC MIXED was used to test the differences in neonicotinoid concentrations in soil with sampling period (pre- or post-plant) as a fixed effect and field as a random effect. PROC CORR was used to test the correlation between the total neonicotinoid concentration in soil and in standing water within fields.

## Results

### Neonicotinoid residues in water

The mean (± SE) neonicotinoid concentration measured in water samples is presented in Table 6. Clothianidin was detected above the LOD in 100% of the 76 samples collected during the 9week sampling period and thiamethoxam was detected in 98.7% (75 out of 76) of the samples. Clothianidin residues were generally higher than those for thiamethoxam (maximum of 43.60 versus 16.50 ng/mL, and mean of 2.28 versus 1.12 ng/mL, for clothianidin and thiamethoxam, respectively).

**Table 6:** Mean (± SE) of total^1^neonicotinoid concentration (ng/mL) measured in water sampled within or in close proximity (≤ 100 m) of maize fields before and after planting in Ontario in 2013.

Sample category		Pre-plant			No. weeks post-planting								
					1–3			4–5			6–7		
	n	Mean	n	SE	Mean	n	SE	Mean	n	SE	Mean	n	SE
Puddle within field	27	1.89	12	0.45	8.72	4	4.26	11.07	5	8.34	3.50	6	1.39
Puddle outside field	21	4.02	6	2.79	1.64	6	0.64	2.32	6	0.59	2.51	3	0.64
Ditch	14	7.54	3	3.54	1.45	6	0.60	2.63	4	1.36	4.63	1	0.00
Drain	7	.	.	.	0.84	5	0.37	3.17	2	3.05	.	.	.
Apiary	7	0.57	3	0.16	0.97	2	0.20	0.67	1	0.00	1.89	1	0.00

^1^Total of clothianidin and thiamethoxam

**Fig 3:**
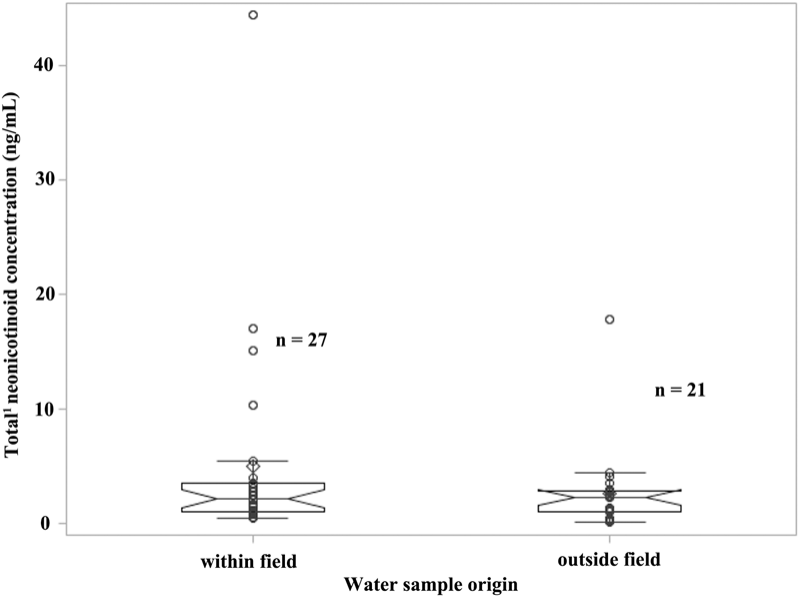
Scatter distribution and notched boxplot of water samples collected from the puddles within/around commercial maize fields, ON, 2013. (^1^Total of clothianidin and thiamethoxam).

Of the 76 water samples taken, 86.8% (66 out of 76) of the samples had total neonicotinoid residue concentrations lower than 5 ng/mL, 6.3% (4 out of 76) of samples had concentrations between 5 and 10 ng/mL, and 7.9% (6 out of 76) of samples above 10 ng/mL. The highest total neonicotinoid concentration measured was 44.38 ng/mL from a puddle within the field in week 4–5 after planting (Fig. 3). The highest total neonicotinoid concentration measured in puddle outside the field, ditch and drain was 17.83, 12.25 and 6.21 ng/mL, respectively.

### Temporal dynamics of residues in water

**Fig 4:**
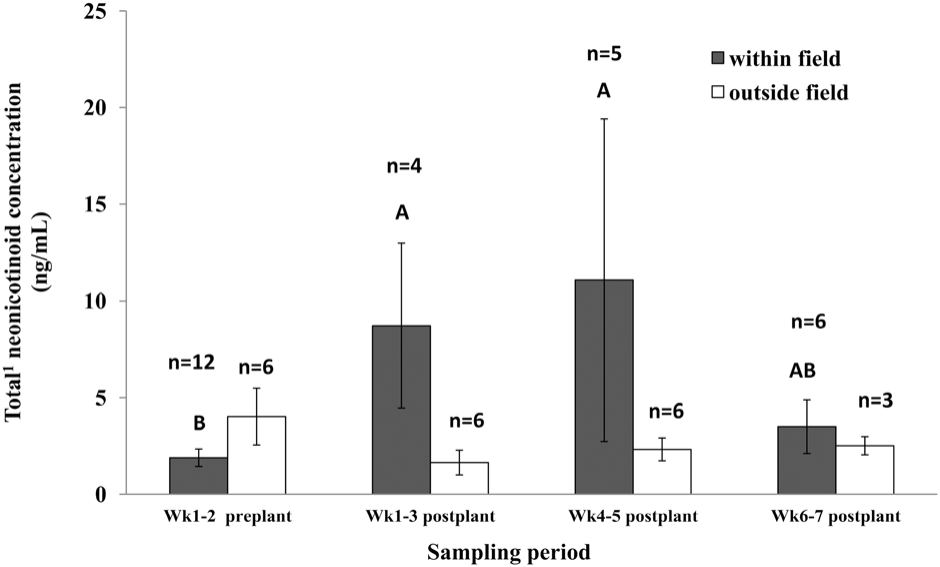
Temporal values of total neonicotinoid concentration (± SEM) for water collected within maize fields compared to samples collected from the puddles outside but in close proximity to these fields. Different letters indicate significant differences in mean neonicotinoid concentration between sampling periods for water sampled within field. (SAS PROC mixed PDIFF, *p* = 0.05). (^1^Total of clothianidin and thiamethoxam).

For water collected from within maize fields, total neonicotinoid concentration increased sharply after maize planting. The concentration in water during weeks 1–3 and weeks 4–5 after planting was 4.6 times higher and 5.9 times, respectively, higher compared with weeks 1–2 before planting (Fig. 4). The concentration in water decreased at weeks 6–7 after plant, and returned to values similar to those measured at the beginning of the sampling period. In contrast, for water samples collected outside of the maize fields, neonicotinoid residues remained constant during the 9-wk period. The concentrations of total neonicotinoids in water sampled from puddles within fields in weeks 1–3, and weeks 4–5 after planting tended to be higher than those collected during the same period from around the field (weeks 1–3, *p =* 0.0861; weeks 4–5, *p* = 0.0572) (Table 7).

**Table 7:** Comparison of total^1^ neonicotinoid concentration between puddles of standing water within and those outside maize fields collected in the same sampling period.

Sampling period	*F*	*df*_*1*_	*df*_*2*_	*p*
Pre-plant	0.37	1	13	0.5524
No. weeks post-planting				
1–3	3.50	1	12	0.0861
4–5	6.06	1	5	0.0572
6–7	0.07	1	7	0.7980

^1^Total of clothianidin and thiamethoxam

## Neonicotinoid concentrations in soil

Multi-residue analysis of the two soil samples collected to provide analytical blanks from the conservation forest each revealed detectable clothianidin (mean 0.03 and 0.11 ng/g), but no thiamethoxam (Table 3). One of the samples contained imidacloprid at the limit of detection (0.01 ng/g). Both samples also contained residues of the herbicide atrazine commonly used in maize fields.

**Fig 5:**
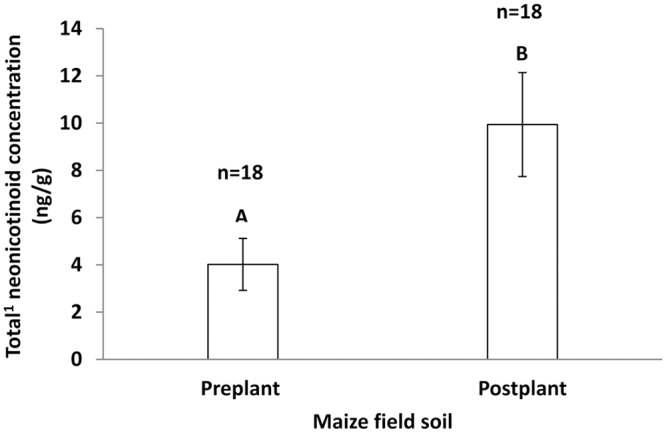
Concentration (± SEM) of neonicotinoid residues in soils, sampled from commercial maize fields, taken pre- and post-planting in ON, 2013. Bars with different letters are significantly different (SAS PROC mixed PDIFF, *p* = 0.05). (^1^Total of clothianidin and thiamethoxam).

The mean neonicotinoid concentration found in the soil sampled to the 5 cm depth within fields after planting was approximately 2.5 times that found in soil sampled immediately before planting (*F* = 9.00, *df*_*1*_ = 1, *df*_*2*_ = 17, *p* = 0.008) (Fig. 5). There were no significant correlations found between soil concentration of neonicotinoid residues determined before or after planting and subsequent concentrations determined in surface water for samples collected within maize fields (Table 8).

**Table 8:** Correlation between total^1^ neonicotinoid concentration in surface soil and standing water within fields.

Water	Soil	Pearson correlation coefficient (r)	*n*	*p*
Pre-plant	Pre-plant	0.0887	12	0.7840
1–3 weeks post-plant	Post-plant	0.5437	4	0.4563
4–5 weeks post-plant	Post-plant	0.0442	5	0.9437
6–7 weeks post-plant	Post-plant	0.5487	6	0.2595

^1^Total of clothianidin and thiamethoxam

### Neonicotinoid insecticides in drifted snow banks around maize fields

For the composite sample taken from the entire vertical profile of the first snow drift, the concentration of all neonicotinoids analysed in the melted snow was below or at the detection limit except for traces of thiacloprid (0.01 ng/mL) (Table 4). Quantifiable residues of clothianidin (0.16 and 0.20 ng/mL) were found in samples taken from the drift stratum containing the most wind-scoured soil as determined visually. Quantifiable levels of the herbicides atrazine and metolachlor were also found in all samples.

## Discussion

These data were collected in a crop season typical of the region. All water samples contained detectable levels of neonicotinoid residues. Water samples collected from within maize fields had higher concentrations than those collected from outside the fields after planting (marginally). While we only sampled during the period from 1–2 weeks before to approximately 7 weeks after planting, neonicotinoid concentrations in water samples from within fields were comparable to those outside fields before planting. They were greater for up to 5 weeks after soils were recharged with insecticide after planting, and then comparable thereafter. For samples collected from within maize fields, only 4 had neonicotinoid concentrations higher than 10 ng/mL. All of these were taken from standing puddles within fields between 1–6 wk after planting following a heavy rain event. The 3 samples with the highest concentrations (15–44 ng/mL) were all from standing puddles associated with post-planting rain events within maize fields taken between 1 and 4 weeks after planting. These results suggest that the greatest exposure for non-target organisms to neonicotinoid residues in water within maize fields occurred during the first 5 weeks after planting and most often after a rain event.

We consider four main sources for neonicotinoid residues contaminating puddles within maize fields: carryover soil residues from previous applications, spilled seed, planted seed, and contaminated fugitive planter dust on the soil surface. Regarding carryover, soil samples collected in the field before planting contained similar levels of carryover residues as the levels of residues measured in the water. This was true for water collected within and outside of the fields before planting, suggesting that soil residue levels might be predictive of those in surface water in and around maize fields before and after the sampling period. However we found no correlation between levels found in water to those found in soil during the period of sampling, with the exception of a few fields that had a higher rate of insecticide introduced either by application rate on the seed or due to replanting; water samples from these fields tended to have higher neonicotinoid concentrations. These data may suggest a more-or-less equilibrium state of association between residues in the soil and those in water in contact with this soil for most of the year. A spike in contamination occurred after soils were re-charged with new insecticide at planting. The most acute source of contamination would likely be puddles forming in areas of the field where treated seeds were spilled and remain on the surface. Goulson [63] reports these exposed seeds as potential sources for acute bird toxicity and birds are much more sensitive to neonicotinoids than most invertebrates. The agricultural industry and regulatory agencies are aware of this exposure risk and require that best management practices be followed to minimize this risk, however continued vigilance is required to minimize the occurrence of exposed treated seed on the soil surface [64–66]. Vacuum-planter exhaust dust [33] after it settles on the soil surface is another potential source for surface water contamination. We measured approximately 1 ng/cm^2^ of total neonicotinoid active ingredient settling on soil surfaces during planting of treated maize using vacuum planters in our own field experiments (data not shown). We also measured 2.5 times the amount of neonicotinoid residues in soil grab samples taken immediately before compared with immediately following planting. These were randomly taken, while avoiding newly seeded rows suggesting that the increase in insecticide residues at the soil surface was a direct result of corn planting, including dust from planter exhaust, large abraded seed treatment particles from seed outlet and from other openings of the planter. Finally, the direct contribution after planting to residues in surface water from insecticide on the seeds is uncertain. Conceivably this could be the largest single source of neonicotinoids to contaminate surface water within fields of maize shortly after planting but little is known about their movement in the soil profile during this period.

Neonicotinoid concentrations (2.41 ng/mL) in water samples collected from around maize fields were approximately 13 fold greater than the mean level of 0.185 ng/g for total neonicotinoids reported for water from wetlands of the Canada Prairie pothole region associated with canola production during the crop growing period [47]. The two regions differ substantially in climate, agronomy and size. The prairies have colder winters, generally less rainfall, and cultivate more canola and small grains in rotation than in Ontario. Therefore neonicotinoid insecticides are used less frequently in the rotation, and mainly for canola production. In contrast, Ontario, where maize is produced, is a region experiencing warmer winters, more precipitation, and more frequent use of neonicotinoid seed treatments on several crops in addition to maize. Our results were also much higher than the maximum 0.257 ng/mL for clothianidin and 0.185 ng/mL for thiamethoxam detected in streams in a high corn and soybean producing region in the United States [56]. We suggest that our samples were taken closer to the time and place of application and subject to much less dilution and movement. Spray drift, surface or subsurface movement of water [67] and perhaps wind movement of treated seeds [47] contribute to the movement of neonicotinoid residues from farmland into waters. Our results support that wind erosion of contaminated soil is a potential contributor to off-target residues in soil [68] and water [47]. We believe our data from snow drifts, and from a non-agricultural soil, combined with the detection of common agricultural herbicides, which are persistent and also known to move in soil dust [69], collectively support this proposal.

Bees often use standing water as a foraging resource, and can collect 44 mg (approximately 44μL) of water during each water-collecting flight [53]. For our worst case, (44.38 ng/mL in a puddle of water in a treated maize field shortly after planting following a rain event), each water foraging bee could collect up to 1.95 ng of neonicotinoid active ingredient in one flight. Similarly if, a bee consumed about 11 μL of water daily at 35°C [54], it could consume up to 0.49 ng of neonicotinoid daily, if that was the only water available. These values are about ten-fold less than the acute oral LD_50_s of 3.8 and 5.0 ng/bee reported for honey bee, for clothianidin and thiamethoxam, respectively [35,36]. In addition, we do not know what portion this water might contribute to the total consumed or collected by a honey bee. The majority of water samples tested, whether originating from within or from outside maize fields, had concentrations less than 5 ng/mL, about 100 fold less than the acute oral LD_50_ used in the example above. These data suggest that standing water in and around the commercial fields of maize were unlikely contributors to acute toxicity to honey bees.

Sublethal effects of neonicotinoid insecticides on non-target species are the subject of much speculation [70,71]. However, for honey bees, the chronic (10 days) no observed effect concentration (NOEC) of clothianidin, thiamethoxam and imidacloprid is in the order of 10 ng/g [35–37,72]. The NOEC of imidacloprid to bumble bees was <2.5 ng/g for reproduction [72]. A sublethal dose of thiamethoxam at 1.34 ng/bee decreased foraging success and survival in honey bees [73]. Clothianidin at 0.5 ng/bee resulted in adverse effects on bee foraging [35,74]. Chronic exposure of imidacloprid at 16 ng/g, or clothianidin at 17 ng/g reduced queen survival, worker movement, colony consumption, and colony weight in *Bombus impatiens* [75]. Only one of the 76 samples we collected might result in an exposure approaching 0.5 ng clothianidin/bee. However, the remaining samples resulted in a potential maximum dose exposure 1/10^th^ that on a daily basis. These data suggest that the standing water in and around the commercial fields of maize sampled were unlikely contributors to sublethal effects on honey bees. However, we speculate that rain water puddles in fields of treated maize in the first weeks after planting may be a source of greater exposures.

Broadly, exposure to neonicotinoid insecticide residues would be greatly reduced if they were used more prescriptively than is currently the case. If most of the seed-applied neonicotinoids are targeted to the seed zone and remain associated with the sub-surface soil solution, based on our data, further limiting the amount of fugitive planter dust landing on the soil surface and limiting the number of exposed treated seeds remaining on the field surface are two practices that would reduce potential acute exposure to contaminated water available to foraging invertebrates within maize fields. These data are broadly informative to risk assessment models for other non-target organisms exposed to standing water in a maize agroecosystem. However, more field data are urgently needed to address the significant data gaps to inform tier three or greater [76] models for exposure risk assessment for non-target invertebrates to neonicotinoids in water; and we report these to assist ecotoxicologists in this urgent task.

## Supporting Information

S1 Table GPS coordinates of experimental fields, beeyards, drift snow and blank soil samples (XLSX)

S2 Table Determination of 7 neonicotinoid residues (ng/mL) in water sampled within or in close proximity (≤ 100 m) of maize fields before and after planting in Ontario in 2013. (XLSX)

S3 Table Summary of means for all water sample types collected, arranged by simple comparisons. (XLSX)

S4 Table Determination of 7 neonicotinoids and 3 herbicide residues (ng/g; dry weight) using QuEChERS sample preparation and LC-ESI(+)-MS/MS analysis of two soil samples collected from a conservation forest of old growth Carolinian species. Clear Creek Conservation Area, Chatham-Kent, ON 2013 (full data with subsamples).
(XLSX)

S5 Table Determination of 7 neonicotinoid insecticide and 3 herbicide residues (ng/g; dry weight) using QuEChERS sample preparation and LC-ESI(+)-MS/MS analysis of field soil in Ontario in 2013. (XLSX)

S6 Table Determination of 7 neonicotinoid insecticide and 3 herbicide residues (ng/ml of melted snow) using QuEChERS sample preparation and LC-ESI(+)-MS/MS analysis in three snow drift samples collected in January 2014 from the edge of two harvested maize fields near Ridgetown, ON (full data with subsamples). (XLSX)
